# Quantitative and Qualitative Radiological Assessment of Sarcopenia and Cachexia in Cancer Patients: A Systematic Review

**DOI:** 10.3390/jpm14030243

**Published:** 2024-02-24

**Authors:** Sveva Mortellaro, Sonia Triggiani, Federica Mascaretti, Micol Galloni, Ornella Garrone, Gianpaolo Carrafiello, Michele Ghidini

**Affiliations:** 1 Postgraduate School in Radiodiagnostics, Università degli Studi di Milano, 20122 Milan, Italy; sveva.mortellaro@unimi.it (S.M.); sonia.triggiani@unimi.it (S.T.); 2 Center for Prevention and Diagnosis of Celiac Disease, Gastroenterology and Endoscopy Unit, Fondazione IRCCS Ca’ Granda Ospedale Maggiore Policlinico, 20122 Milan, Italy; federica.mascaretti@policlinico.mi.it; 3 Cryovis s.r.l, 20122 Milan, Italy; gallonimicol2@gmail.com; 4 Oncology Unit, Fondazione IRCCS Ca’ Granda Ospedale Maggiore Policlinico, 20122 Milan, Italy; ornella.garrone@policlinico.mi.it; 5 Operative Unit of Radiology, Fondazione IRCCS Ca’ Granda Ospedale Maggiore Policlinico, 20122 Milan, Italy; gianpaolo.carrafiello@unimi.it; 6 Department of Oncology and Emato-Oncology, Università degli Studi di Milano, 20122 Milan, Italy

**Keywords:** sarcopenia, cachexia, radiological assessment, BIA, DEXA, CT scan, MRI

## Abstract

Sarcopenia, an extremely common condition in cancer patients, is described as a progressive and generalized musculoskeletal disorder that is associated with an increased likelihood of adverse outcomes, including falls, fractures, physical disability, and mortality. By contrast, cachexia is defined as a syndrome characterized by weight loss with the concomitant loss of muscle and/or fat mass. Cancer cachexia leads to functional impairment, reduced physical performance, and decreased survival, and is often accompanied by cancer progression and reduced response to therapy. The literature states that cancer patients with cachexia or sarcopenia have many more complications than patients without these conditions. The interplay between physiologic sarcopenia and cancer cachexia is, in part, responsible for the complexity of studying wasting disorders in the cancer population, particularly in the geriatric population. For these reasons, a comprehensive assessment of the body composition and physical function of these patients is necessary. There are several modalities adapted to measure skeletal muscle mass, such as dual-energy X-ray absorptiometry (DEXA), bioelectrical impedance analysis (BIA), computed tomography (CT), magnetic resonance imaging (MRI), and ultrasound (US). The gold standard for the measurement of quantitative and qualitative changes in body composition in patients with cancer is the analysis of tissue density using a CT scan. However, this technique remains poorly implemented in clinical practice because of the use of ionizing radiation. Similarly, DEXA, MRI, and US have been proposed, but their use is limited. In this review, we present and compare the imaging techniques that have been developed so far for the nutritional assessment of cancer patients.

## 1. Introduction

Sarcopenia and cachexia are two conditions associated with negative health outcomes. They share common characteristics, including skeletal muscle wasting, inflammation, weakness, and fatigue, but they differ in other aspects, aetiologias, and treatments [1,2]. Sarcopenia is a progressive and generalized skeletal muscle disorder that leads to an increased risk of falls (with the consequent increased probability of fractures), physical disability, poor quality of life, and mortality [3,4]. Sarcopenia can develop due to insufficient energy or protein intake but a sedentary lifestyle or inability to move can also contribute to the condition [4]. The diagnosis of sarcopenia is confirmed by the presence of low muscle quantity or quality and can be detected using a validated screening test called SARC-F [5]. When a patient shows low muscle strength, low muscle quantity/quality, and low physical performance, sarcopenia is considered severe [4]. There are two types of sarcopenia: primary and secondary. The first is age-related and has no specific causes. The secondary is caused by a systemic inflammatory condition, which is typical of tumor pathologies [4].

The diagnosis of sarcopenia depends on the measurement of muscle strength, muscle quantity, and physical performance [4,6]. Muscle strength can be measured with a calibrated handheld dynamometer, which is a simple and inexpensive instrument. Low muscle strength can increase the length of hospital stay and decrease the ability to move, leading to a poor quality of life [4,6]. Muscle strength can be estimated through various tests: magnetic resonance imaging (MRI), computed tomography (CT), dual-energy X-ray absorptiometry (DEXA), and bioelectrical impedance analysis (BIA). The third method of measuring sarcopenia is physical performance, which is defined as the ability to independently perform daily physical activities [7]. The most commonly used tests for physical performance are gait speed and a timed 400 m walk, the Short Physical Performance Battery, the Timed Up and Go test, and the chair stand test (moving five times from sitting to standing) [6]. Sarcopenia is associated with malnutrition because, especially in cancer patients, reduced food intake can occur due to a loss of appetite, therapy-related side effects (e.g., diarrhea and vomiting), and increased energy demands given by the inflammatory process [4]. This condition of sarcopenia leads the cancer patient to depression, adverse clinical outcomes, severe chemotherapeutic toxicity, and, in severe cases, the inability to continue with oncological therapies [1]. Cachexia is a complex metabolic process that can develop in various diseases (e.g., cancer, heart failure, acquired immune deficiency syndrome, renal failure) [8]. Cancer cachexia is a multifactorial condition characterized by a continuous loss of skeletal muscle mass (with or without the loss of fat mass), leading to a progressive functional impairment that cannot be completely resolved by conventional nutritional support [9]. The key manifestation of cancer cachexia is loss of muscle mass; however, it is a systemic condition in which anorexia, inflammation, insulin resistance, and muscle proteolysis are frequently observed and may affect multiple organs [10,11].

Cachexia is primarily determined by a negative energy balance. The main contributing factors are reduced appetite due to alterations in taste and smell, resulting in a decrease in energy intake of approximately 300–500 kcal/day, and metabolic changes such as hypermetabolism generated by a pro-inflammatory environment [8,12,13]. Cachexia is particularly prevalent in tumors of the upper gastrointestinal tract and the lung, affecting 83–85% of patients with gastric and pancreatic cancers and 60% of patients with lung cancer [8]. In 2011, Fearon et al. defined and classified cancer cachexia into three stages (not all patients go through all the stages): precachexia, cachexia, and refractory cachexia. Precachexia anticipates cachexia and is characterized by mild and involuntary weight loss (≤5%), anorexia, and metabolic abnormalities [9,10]. Once identified, it should allow early treatment before worsening into cachexia and, subsequently, into refractory cachexia. This is an advanced stage in which there is a rapid progression of cancer non-responsive to therapies [9,10]. However, cancer cachexia can occur in the absence of fat/weight loss and takes the name of ‘hidden cachexia’, in which it is fundamental to measure muscle loss and not only evaluate weight loss [2,11] (Table 1).

**Figure 1:**
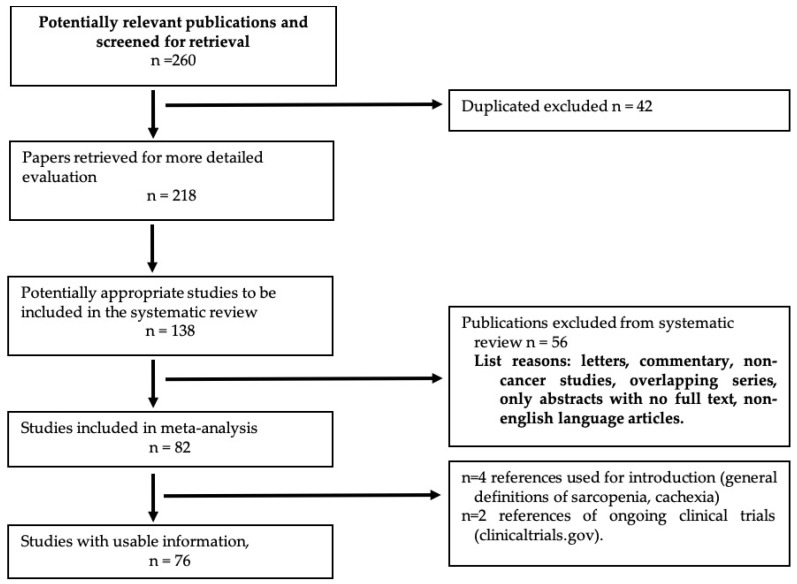
PRISMA flow chart. Overview of trial search and selection.

In 2021, the European Society of Medical Oncology (ESMO) provided clinical practice guidelines for patients with cancer cachexia. Cachexia is defined as a “disease-related malnutrition based on the Global Leadership Initiative on Malnutrition (GLIM) definition and the presence of systemic inflammation” [12]. The same guidelines stated that the treatment of patients with cachexia should be based on a multimodal approach, ensuring adequate energy and nutrient intake, minimizing catabolic alterations, supporting muscle training, and providing psychological support [12]. Figure 1 shows a PRISMA flow chart for the selection of studies included in the systematic review.

**Figure 2:**
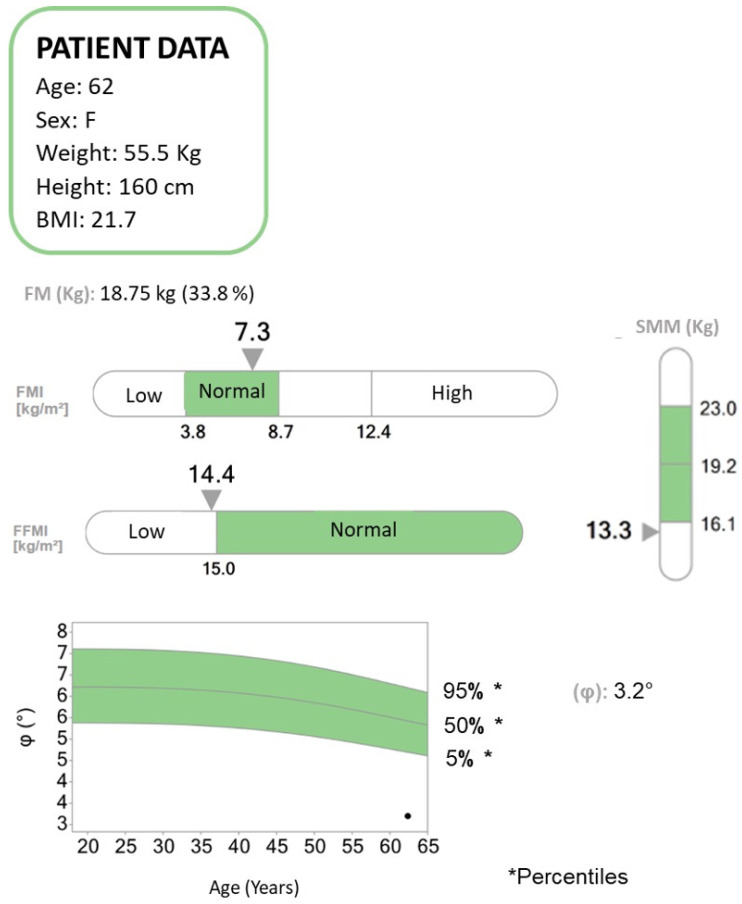
Bioelectrical impedance analysis (BIA) report concerning a patient diagnosed with pancreatic neoplasia and liver metastases undergoing chemotherapy. BIA is a method to assess the body composition of patients in terms of fat mass (FM), fat-free mass (FFM), and skeletal muscle mass (SMM). The patient suffered a weight loss of approximately 20 kg in the past year. According to the body mass index (BMI), a value expressed as the ratio of weight to the square of the height, the patient falls within the normal range. From the BIA analysis, it emerges that the FM is within normal limits, while the FFM and the SMM are below the normal range. The phase angle (PhA, ϕ) is low. The PhA has been suggested as an indicator of cell integrity, cell functions, and water distribution. Higher values reflect significant quantities of intact cell membranes, while lower PhA values suggest decreased cell integrity. PhA decreases in pathologies such as cancer and states of inflammation and malnutrition. It also plays an important prognostic role in various pathologies and has been associated with mortality and sarcopenia.

There are several modalities to define a patient’s body composition. Anthropometric measurements (e.g., BMI, calf circumference, and skinfold thickness) are cheap, easily available, and mostly used in clinical practice [13,14,15,16]. However, they may not provide precise assessments of different body compartments. For a more accurate evaluation of skeletal muscle mass in sarcopenia and cachexia conditions [16,17], MRI and CT are considered gold standards but are not commonly used in primary clinical practice due to their high costs, lack of portability, and the need for highly trained personnel [4,18,19]. DEXA is a technique to determine muscle quantity through a low-dose radiation technique [4,19]. BIA is relatively cheap, portable, and easy-to-use and it could be a valid alternative to CT, DEXA, and MRIs in clinical practice (Figure 2) [20].

## 2. Traditional Body Composition Indicators

There are several methods to analyze the body composition of patients. Among the most widely used are traditional indicators of body composition such as BMI [11,21,22,23,24], body mass index, BIA, bioelectrical impedance analysis [25,26], and SFT (skinfold thickness) [27], which are very practical and easy to apply, but cannot be considered universal indicators because they may underestimate body fat in some ethnic groups and others that physiologically tend to have higher fat percentages, such as Asians, Indians, and the elderly, gender, sex or fat distribution in patients [22,28,29,30,31]. For these reasons, currently, the most widely used methods in clinical practice to assess the body mass of patients are instead quantitative indicators of body composition, namely CT, MRI, and positron emission tomography (PET) [32]. These imaging modalities are usually used in the diagnosis and follow up of cancer patients, so they can also be applied to assess the body composition of these patients. To date, however, it remains an open question which of these methods is more accurate for assessing body composition, given the future challenges and opportunities offered by each modality [32,33,34].

## 3. Quantitative Imaging Markers of Body Composition

### 3.1. Computed Tomography

**Figure 3:**
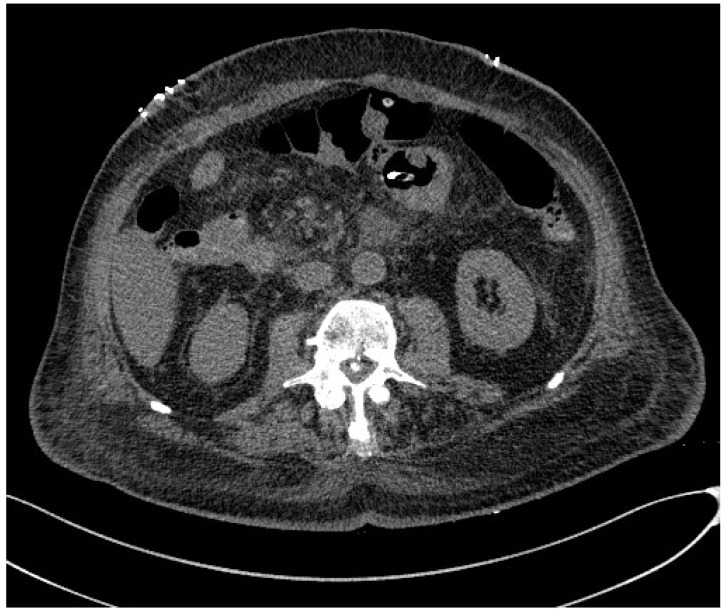
Non-contrast-enhanced CT scan of a sarcopenic patient with pancreatic carcinoma.

**Figure 4:**
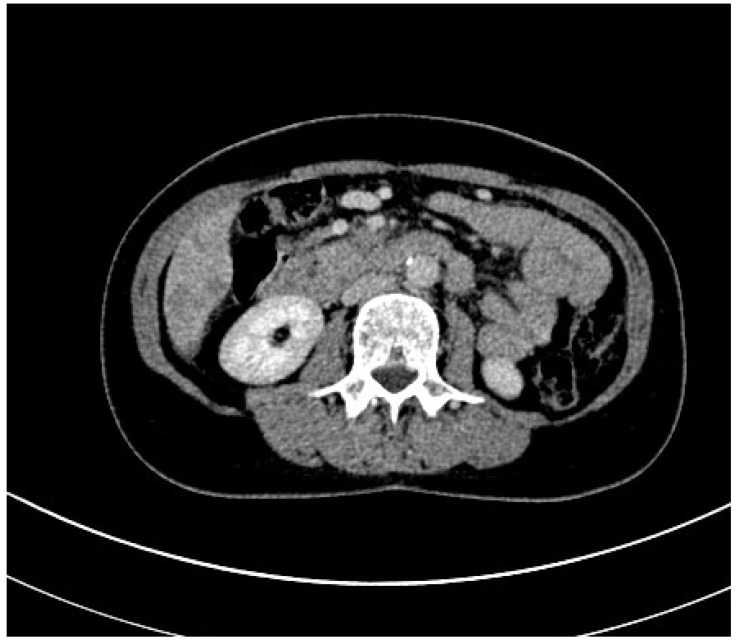
Contrast-enhanced CT scan of a sarcopenic patient with pancreatic carcinoma.

Computed tomography (CT) is an imaging modality that uses X-rays to construct cross-sectional images (‘slices’) of the body. Cross-sections are reconstructed from measurements of the attenuation coefficients of X-ray beams passing through the volume of the object being studied [35]. CT is based on the fundamental principle that the density of the tissue crossed by the X-ray beam can be measured by calculating the attenuation coefficient [35,36]. Using this principle, CT allows the various densities of the body to be reconstructed by means of a two-dimensional cross-section perpendicular to the axis of the acquisition system. CT imaging technology has been used in clinical settings since the 1970s and, over time, has evolved to provide higher speed, better image quality, and lower radiation doses [35,36,37]. In newer versions of CT, multiple tubes and detectors (i.e., a multidetector CT) are integrated into a ring configuration and are rotated around the patient while the patient is moved on a sliding bed. The results are a full 360-degree scan that could, in theory, extend from head to toe, with the actual body part scanned depending on the clinical question asked [35,36,37,38]. Furthermore, new advances in automated CT scan segmentation and deep learning have opened the door to the implementation of CT body composition as an innovative tool for assessing health and disease risk. In this regard, a study published in 2016 states that it is important for muscle measurements to be performed in non-contrast sequences, as muscle attenuation values tend to increase after an intravenous contrast medium injection. This could distort the measurements. In addition, measurements should be taken at the level of the third to fourth lumbar vertebrae [39] (Figure 3 and Figure 4). The function that allows CT to be considered reliable in the assessment of body mass is the attenuation of X-ray beams as they pass through various tissues, expressed in Hounsfield units (HUs), which then allows two-dimensional images to be reconstructed [35,36,37,38,39,40]. Thus, in the context of neoplastic cachexia, CT scans can quantify whole-body, regional, and individual volume changes in skeletal muscle and visceral adipose tissue (VAT) [41]. It can also determine the regional and individual distribution of fat deposits. A CT can be further applied to assess myosteatosis (fat infiltration into skeletal muscle) by measuring the mean radiodensity of muscle tissue, which is known to be linearly dependent on the fraction of the skeletal muscle fat and is linked to poorer performance in cancer patients [40,41]. In a recent systematic review, eight studies showed that the reduction in muscle mass was mainly detected by CT body composition analysis, with a high number of patients misclassified according to BMI [41]. Body composition CT detects sarcopenia at a rate of 27.3–66.7% higher than the detection of malnutrition by BMI [41,42]. A further meta-analysis emphasized the importance of the assessment of myosteatosis on a CT scan, identifying that patients with lymphoma, gynaecological, renal, pancreatic, hepatocellular, gastroesophageal, and colorectal cancer who had a higher myosteatosis on a CT scan had worse overall survival [43]. In another recent study, including head and neck tumors, it was stated that patients with a low VAT volume and high VAT HU had significantly worse progression-free survival and distant failure-free survival than those with a high VAT volume and low VAT HU [41]. Thus, VAT volume and CT attenuation were significantly related to disease progression-free survival and distant failure-free survival in such patients [41]. However, CT scanning has some limitations that are mainly related to cost and radiation exposure, which makes it difficult to obtain images for the sole purpose of measuring body composition. The nature of the CT scanner limits its portability, which is an important consideration when designing larger-scale studies [44].

### 3.2. Positron Emission Tomography (PET)

Positron emission tomography (PET) is an excellent non-invasive imaging procedure with a wide range of clinical and research applications. It involves the intravenous injection of a radiopharmaceutical followed by the measurement of its binding and uptake in tissues. The radiopharmaceutical mainly used in oncology is 2-[18F] fluoro-2-deoxy-D-glucose (FDG), which measures glucose uptake, a surrogate marker of glycolysis that is a well-characterized hallmark of cancer cells [45]. Indeed, it detects the metabolic activity of tumors by quantifying the standardized uptake value (SUV) [45]. FDG-PET, unlike other imaging modalities, allows the quantification of tumor metabolism; higher glucose uptake in tumors correlates positively with more aggressive disease and worse prognostic outcomes. Moreover, unlike CT, FDG-PET can identify and distinguish white and brown adipose tissue (BAT) [46,47]. In fact, in a recent study, BAT volume, as assessed by routine PET/CT, was shown to be a predictor of tumor recurrence/mortality in cancer patients, independent of other factors that may influence BAT activity, such as sex, age, BMI, or tumor type [48]. The other major advantage of PET is the possibility of combining it with other imaging modalities, such as CT or MRI, to gather more information, including anatomical information. In particular, PET scans are generally performed simultaneously with CT to correlate radiotracer activity with anatomy and to correct the PET signal for the attenuation of tissue measured in HU by CT. It should be mentioned, however, that measuring body composition using CT and PET simultaneously is more limited [49]. However, in a recent study on lymphoma and its different distributions in the two sexes in this disease, it was seen that with FDG-PET/TC, it is possible to predict, based on the different uptake of visceral fat, the outcomes in patients with DLBCL in a sex-specific manner. It is, therefore, a very useful system in the follow up of cancer patients [49]. PET-MRI, on the other hand, is less available and used to date than PET-CT. MRI does not use ionizing radiation but magnetic fields and radio frequencies. It is also a multiparametric and multiplanar imaging technique, allowing images to be acquired in the sagittal, dorsal, or transverse planes without moving the patient [50,51]. PET/MRI, and thus the fusion of these two methods, is useful in staging and re-staging tumors, as suggested by a recent update published in the literature on head and neck cancers, according to which PET/MRI has the same performance as PET/CT [52]. Moreover, it has a role in quantifying the metabolic rate of glucose, lipid content, and perfusion in brown adipose tissue (BAT). However, due to its high cost, its use is not yet widespread [52]. In conclusion, PET, like other methods, has some limitations that are mainly related to patient exposure to both radiopharmaceuticals and ionizing radiation, which can make it problematic to perform studies in healthy populations [53].

### 3.3. Dual-Energy X-ray Absorptiometry (DEXA)

Dual-energy X-ray absorptiometry (DEXA) is an additional available technique to quantify different tissues in patients. It is a widely accessible, fast, and inexpensive two-dimensional projection technique suitable for assessing body composition by estimating body fat, muscle tissue, and bone mineral density [53,54]. In the context of neoplastic cachexia, DEXA has been used to measure the body and regional distribution of skeletal muscle and VATS [20,53,55]. There are multiple studies in the literature about its use in breast cancer, neuroendocrine tumors, and, in general, on the assessment of muscle dysfunction in cancer patients [55,56,57]. In a recent study of breast cancer patients who developed arm lymphedema after lymph node emptying, DEXA was seen to give excellent results when used to assess the extracellular volume of the limb, resulting in hypertrophy and muscle dysfunction [56]. Another study on patients with neuroendocrine tumors of gastro–entero–pancreatic origin (GEP-NET) showed how DEXA can monitor changes in fat and muscle composition in these patients, providing indications of likely cancer progression or response to treatment [57].

### 3.4. Magnetic Resonance Imaging (MRI)

MRI has emerged as a powerful diagnostic tool for the radiological assessment of sarcopenia and cachexia in cancer patients. MRI offers several advantages over traditional imaging techniques for assessing muscle quality and quantity in cancer patients. Unlike X-rays or CT scans, MRI does not involve ionizing radiation, making it a safer option for repeated evaluations, especially in cancer patients undergoing multiple treatments. MRI provides an exceptional soft tissue contrast, allowing for the detailed visualization of muscle, fat, and other tissues. With the advent of advanced MRI techniques such as Dixon and chemical shift imaging, it is possible to differentiate between muscle and fat components accurately. One of the key strengths of MRI in assessing sarcopenia and cachexia is its ability to provide qualitative and quantitative data [58]. MRI enables the detailed qualitative assessment of muscle tissue in cancer patients, aiding in the diagnosis and staging of sarcopenia and cachexia. Key qualitative parameters include muscle mass, muscle quality, and muscle symmetry. MRI provides a clear visualization of muscle mass, allowing for the easy identification of muscle atrophy, which is a hallmark of both sarcopenia and cachexia. Muscle loss can be observed as a reduction in the muscle cross-sectional area. As for muscle quality, MRI can distinguish between healthy muscle tissue and muscle infiltrated with fat, fibrosis, or edema. It enables the assessment of muscle quality by identifying the presence of these pathological changes. A bilateral comparison of muscles can help identify asymmetry, which may be indicative of muscle wasting or imbalances due to disease-related factors [59]. A quantitative assessment using MRI goes beyond qualitative observations and provides precise measurements, facilitating the tracking of disease progression and the response to therapeutic interventions. Key quantitative parameters include muscle volume, fat infiltration, muscle strength prediction, and the muscle cross-sectional area. MRI can measure muscle volume by segmenting muscle groups, allowing for the precise quantification of muscle loss or gain over time. This measurement can help assess the progression of sarcopenia and cachexia. MRI can quantitatively evaluate the extent of fat infiltration within muscle tissue. Excessive fat infiltration, known as myosteatosis, is a critical marker of muscle deterioration in cancer patients [60,61]. Some MRI techniques can indirectly estimate muscle strength by evaluating the muscle volume and quality. This information is valuable for predicting functional limitations and assessing the risk of falls or injuries. MRI can provide quantitative data on the muscle cross-sectional area at various levels of the body, aiding in the monitoring of muscle atrophy and the effectiveness of interventions [62]. In 1998, a study validated MRI and CT as accurate methods for estimating appendicular adipose tissue in vivo without skeletal muscle and interstitial and subcutaneous adipose tissue [63]. With regard to the field of oncology, brown adipose tissue (BAT) has recently been recognized as a significant contributor to the rapid weight loss and malnutrition observed in malignancies, and the results of a study in a mouse model illustrated that the use of MRI can detect and measure BAT during pancreatic cancer progression [64]. With a retrospective analysis of children aged 1–15 years affected by neuroblastoma, Ritz et al. decided to measure the total psoas muscle area (tPMA) on cross-sectional imaging as sarcopenia is an independent marker for poor post-surgical outcomes. CT and MRI scans were both used to measure the psoas muscle area at L3-L4 and L4-L5 lumbar disc levels. They concluded that the evaluation of tPMA using pre-surgical cross-sectional imaging is a biomarker for enhanced risk evaluation in pediatric neuroblastoma cases [65]. The role of the 3T MRI scanner was investigated for the first time by Auckland’s Cancer Cachexia Evaluating Resistance Training (ACCeRT) based on the assumption that, from a physical point of view, the signal-to-noise ratio is doubled to the higher field strength of the 3T scanner. Studying a small cohort of non-small cell lung cancer (NSCLC) patients, they concluded that there was a difference in muscle gained by gender, but more studies are needed with the aim to target anabolic muscle pathways in patients with end-stage or refractory cachexia [66]. MRI has the capability not only to quantify the cross-sectional dimensions of muscle and its mass but also to differentiate various tissue categories, such as muscle and subcutaneous adipose tissue. An investigation employed a statistical clustering approach (k-means) on MR scans of the quadriceps in young and elderly healthy males and females with cancer to objectively distinguish the contractile and non-contractile tissue components [67]. They concluded that K-means provide an objective differentiation of contractile and non-contractile tissue elements. Females diagnosed with upper gastrointestinal (GI) cancer exhibit notable fat infiltration across entire muscle groups, and this observation remains consistent when accounting for age [67]. MRI has proven to be an invaluable tool for the qualitative and quantitative radiological assessment of sarcopenia and cachexia in cancer patients. Its ability to offer detailed insights into muscle mass, quality, and fat infiltration provides clinicians with critical information for diagnosis, prognosis, and treatment planning. By combining both qualitative and quantitative data, MRI helps in monitoring the progression of these conditions and evaluating their response to therapeutic interventions, ultimately contributing to better outcomes and improved quality of life for cancer patients. As technology advances, MRI continues to evolve, promising even more accurate and detailed assessments of these conditions in the future.

### 3.5. Ultrasound (US)

Ultrasound has emerged as a promising and accessible tool for both the qualitative and quantitative radiological assessment of sarcopenia and cachexia due to its non-invasive, cost-effective, real-time, and non-ionizing nature. Ultrasound imaging is particularly valuable in the qualitative evaluation of muscle and in its distinction between muscle and fat tissues, enabling the early detection of these conditions and guiding therapeutic interventions [68]. The quantitative assessment of sarcopenia and cachexia using ultrasound has also gained prominence due to its accuracy and affordability. Ultrasound allows for the precise measurement of muscle dimensions, including the cross-sectional area and thickness. Additionally, advanced techniques such as muscle shear wave elastography can provide quantitative data on muscle stiffness, which is a valuable indicator of muscle health. These quantitative measures can help track changes in muscle mass and composition over time, enabling the early detection of muscle wasting and guiding personalized treatment plans. A recent systematic review highlighted the lack of a standardized technique and cut-off values in different clinical populations, although newly created guidelines reflecting a consensus were published to provide carefully supported practical recommendations for enhancing the reproducibility and validity of skeletal muscle ultrasound (SMUS) by specifying the optimal technique for each muscle site [68,69]. To provide the standardization of ultrasound measurements in 2020, the SARCUS working group discussed four new muscle parameters (microcirculation, volume, contraction potential, and stiffness) besides the already known parameters (cross-section area, pinnation angle, fascicle length, echo intensity, and thickness) [69]. Another study group evaluated the correlation between the Strength, Assistance for Walking, Rise from a Chair, Climb Stairs, and Falls (SARC-F) score and ultrasound-derived muscle thickness in older hospitalized cancer patients, and they observed a moderate inverse relationship and no systematic bias risk between them [70]. Focusing on a selective population of metastatic breast cancer patients, Escriche-Escuder et al. used ultrasound to measure body composition changes after exercise intervention. By analyzing the muscle thickness and echo intensity of the biceps brachii, brachialis, and quadriceps muscles, the study team concluded that ultrasound biomarkers associated with muscle architecture, particularly muscle thickness, showed greater responsiveness than those related to adipose tissue and patient-reported outcomes [71]. Another study group investigated the role of the ultrasound of the rectus femoris muscle in locally advanced head and neck squamous cell carcinoma as a predictor of postoperative complications and decreased overall survival. They concluded that this evaluation helped identify a subset at high risk of 30-day postoperative complications and poorer overall survival, highlighting the individuals who would particularly benefit from targeted “prehabilitation” [72]. The role of ultrasound has been explored in adult patients undergoing treatment for non-small-cell lung cancer, correlating ultrasound biomarkers with the MRI-based percentage of fat, histology, and CT-based muscle density [73]. The researchers compared the results in the study group population with a population of healthy adults and concluded that while ultrasound-based rectus femoris echo intensity exhibited less sensitivity in distinguishing myosteatosis between groups, it remained strongly correlated with MRI-based proton density fat-fraction (PDFF). According to their results, ultrasound measurements can be employed for the bedside assessment of myosteatosis and offer greater diagnostic utility compared to conventional weight evaluations such as BMI [73]. An interesting association between muscle volume and function in patients with cancer-related cachexia was studied by Weber et al., and although muscle volume was reduced, it did not correlate with a loss in function. They evaluated the cross-sectional area (CSA) of the quadriceps femoris muscle by means of MRI scans, and the energy and lipid metabolism of the vastus lateralis muscle was measured by 31P and 1H spectroscopy, studying biopsies of the vastus lateralis muscle; they quantified skeletal muscle fiber size and capillarization, and microcirculation was evaluated by contrast-enhanced ultrasonography (CEUS). So, the preservation of contracting functionality should serve as a justification for shifting away from the common practice of advising rest and, instead, focusing on training the muscles of these patients [74]. Therefore, ultrasound is a valuable imaging modality for the qualitative and quantitative assessment of sarcopenia and cachexia in cancer patients. With ongoing research and the improved standardization of protocols, ultrasound is poised to play a crucial role in the early detection and monitoring of these debilitating conditions, ultimately contributing to better patient outcomes in the field of oncology. Table 2 summarizes the findings for sarcopenia and cachexia using different diagnostic methods, while Table 3 compares different imaging methods in terms of availability, costs, and transportability.

## 4. Discussion

Cancer sarcopenia and cachexia are multi-organ syndromes that bring changes to body composition over time. In daily clinical practice, body composition is often estimated rather than measured. Indeed, anthropometry may be affected by inter and intra-observer variability. Moreover, the routine use of BIA derives fat and fat-free mass values indirectly from hydration status, and its results may be altered in the case of pathological conditions such as ascites and hydrothorax. Differently, medical non-invasive imaging allows a precise measurement of body composition and holds the biggest potential in assessing the changing phenotype of sarcopenia and cachexia during the treatment journey [75]. The gold standard for the evaluation of changes in body composition is the analysis of tissue density using a CT scan at the level of the third lumbar vertebra, despite bone metastases originating from different primary tumors could determine bone changes [76]. This technique allows us to evaluate not only quantitative changes (i.e., sarcopenia) but also qualitative modifications, including myosteatosis [60]. Despite this, CT scans remain poorly implemented in the field of the nutritional assessment of cancer patients, mainly because of the use of ionizing radiation. Similarly, the use of MRI and FDG PET is infrequent due to low repeatability and high costs. Also, DEXA and US have been proposed in order to assess body composition. Despite being less precise than a CT scan and PET, these techniques are inexpensive and easily reproducible. In this direction, the JUMP Research II study aims to evaluate the correlation between the muscle mass (thickness) of the quadriceps assessed in axial sections by ultrasound and the reference muscle mass index (Appendicular Lean Mass (ALM)/height^2^) assessed by DEXA in post-cancer patients. Moreover, among the secondary endpoints, this study correlates the US muscle indexes and the scannographic index of the paravertebral muscles (SMI L3, T12, and T7), studied with artificial intelligence [77]. Similarly, another ongoing study is evaluating the impact of androgen deprivation agents on muscular mass in patients with prostate cancer. Changes in lean body mass (LBM) and fat mass are measured by DEXA, while changes in muscle thickness are determined via ultrasound [78]. This study has some limitations. Non-English language articles and articles whose full text was not available were excluded.

## 5. Conclusions

Increasing evidence supports the use of imaging tools in order to assess the nutritional status of cancer patients, especially in pathological conditions such as sarcopenia and cachexia. CT scan and MRI are favorable approaches that produce high spatial and contrast resolution images, while DEXA and US are more reproducible and inexpensive. Further studies should be conducted in order to generate a comprehensive understanding of the processes involved in cancer patients with sarcopenia and cachexia, with attention to factors such as cancer type, stage, and current therapy. In this direction, the use of AI as a radiological tool may add value and reliability to these techniques.

## Figures and Tables

**Table 1:** Characteristics of cachexia and sarcopenia.

Features	Cachexia	Sarcopenia
Definition	Disease-related malnutrition based on the GLIM definition and the presence of systemic inflammation [12].	Decline in skeletal muscle mass and function, increased risk of falls, physical disability, poor quality of life, and mortality [3,4,9].
Causes	Negative energy balance (increased energy expenditure and decrease in energy intake) and metabolic alterations generated by a pro-inflammatory environment [8,12,13].	Inflammation, reduced physical activity, malnutrition, and direct effects of cancer therapies [4].
Clinical features	Weight loss, decreased skeletal muscle mass, anorexia, and metabolic abnormalities [2,10,11].	Low muscle strength, low muscle quantity/quality, and low physical performance [4,5,6].

Abbreviations: GLIM: Global Leadership Initiative on Malnutrition.

**Table 2:** Findings for sarcopenia and cachexia using different diagnostic methods.

Source	Pathology	Diagnostic Method	Results
Aleixo GFP. et al. [3]	Sarcopenia	CT	- Body composition; - Muscle measurements; - Myosteatosis and muscle mass reduction; - Psoas muscle at the L3–L4 level and total muscles at the L4 level.
Lee JW. et al. [41]	Cachexia		- Increased attenuation of adipose associated with reduced disease progression-free survival in non-contrast images.
Witney TH. et al. [45] Chu K. et al. [48] Jaswal S. et al. [49] Seifert R. et al. [51]	Sarcopenia	FDG-PET	- Metabolic changes that may be present before clinically detectable changes; - Identifies and distinguishes WAT and BAT; - Skeletal muscle biology and measure of insulin resistance.
Chu K. et al. [48]	Cachexia		- Metabolic activity of BAT by measuring FDG uptake along the neck, supraclavicular, mediastinal, and paravertebral regions.
Chianca V. et al. [59]	Sarcopenia Cachexia	MRI	- Qualitative parameters: muscle mass, muscle quality, and muscle symmetry.
Tagliafico AS et al. [61] Han J et al. [60]	Sarcopenia Cachexia		- Quantitative parameters: muscle volume, fat infiltration, muscle strength prediction, and muscle cross-sectional area.
Boutin RD et al. [62]	Sarcopenia		- Estimation of muscle strength, predicting functional limitations, and assessing the risk of falls or injuries.
Zhang Y et al. [64]	Sarcopenia	MRI	- Monitoring the function and volume of BAT in mice with tumors.
Ritz A et al. [65]	Sarcopenia		- Assessing pre-surgical tPMA as a biomarker may serve as an additional means for stratifying the risk in children with neuroblastoma.
Rogers ES et al. [66]	Cachexia		- Difference in muscle gained by gender.
Gray C et al. [67]	Sarcopenia		- K-means objectively distinguishes between contractile and non-contractile tissue components.
Casey P et al. [68]	Sarcopenia	US	- Lack of standardized technique and cut-off values in distinct clinical populations.
Perkisas S et al. [69]	Sarcopenia		- Four new muscle parameters (microcirculation, volume, contraction potential, and stiffness) for standardization.
Gomes TLN et al. [70]	Sarcopenia		- Moderate inverse relationship and no systematic bias risk between SARC-F score and ultrasound-derived muscle thickness.
Escriche-Escuder A et al. [71]	Sarcopenia		- High responsiveness of ultrasound biomarkers associated with muscle architecture of biceps brachii, brachialis, and quadricep muscles in metastatic breast cancer.
Galli A et al. [72]	Cachexia	US	- US of rectus femoris muscle in locally advanced head and neck squamous cell patients helps identify a subset at high risk of 30-day postoperative complications and poorer overall survival
Lortie J et al. [73]	Sarcopenia Cachexia		- US measurements can be employed for the bedside assessment of myosteatosis and offer greater diagnostic utility compared to conventional weight evaluations such as BMI.
Weber MA et al. [74]	Cachexia		- Muscle volume reduction is not correlated to a loss of functionality.

Abbreviations: BAT: brown adipose tissue; BMI: body mass index; CT: computed tomography; FDG-PET: fluoro-2-deoxy-D-glucose positron emission tomography; MRI: magnetic resonance imaging; tPMA: total psoas muscle area; US: ultrasound; WAT: white adipose tissue.

**Table 3:** Comparison between different imaging methods.

	Availability	Costs	Transportability	Examiner Dependency
CT	High	Medium	Not transportable	Independent
FDG-PET	Medium	High	Not transportable	Independent
MRI	Medium	High	Not transportable	Independent
US	High	Low	Transportable	Dependent

Abbreviations: CT: computed tomography; FDG-PET: fluoro-2-deoxy-D-glucose positron emission tomography; MRI: magnetic resonance imaging; US: ultrasound.

## Abbreviations

BAT	brown adipose tissue
BIA	bioelectrical impedance analysis
BMI	body mass index
CT	computed tomography
DEXA	dual-energy X-ray absorptiometry
FDG-PET	fluoro-2-deoxy-D-glucose positron emission tomography
FFM:	fat-free mass
FM	fat mass
HU	Hounsfield units
MRI	magnetic resonance imaging
PhA	phase angle
SMM	skeletal muscle mass
US	ultrasound
VAT	visceral adipose tissue
